# Principles of radiological protection and application of ALARA, ALADA, and ALADAIP: a critical review

**DOI:** 10.1590/1807-3107bor-2025.vol39.014

**Published:** 2025-02-07

**Authors:** Rafael Pereira de MENDONÇA, Carlos ESTRELA, Mike Reis BUENO, Teresa Cristina Alves Silva Gonzalez CARVALHO, Lucas Rodrigues de Araújo ESTRELA, Israel CHILVARQUER

**Affiliations:** (a) Universidade de São Paulo, School of Dentistry, Oral Diagnosis, Dental Radiology, and Imaging Department, São Paulo, Brazil.; (b) Universidade Federal de Goiás - UFG, School of Dentistry, Stomatologic Science Department, Goiânia, GO, Brazil.; (c) CROIF, Diagnostic Imaging Center, Cuiabá, MT, Brazil.; (d) Universidade Estadual Paulista – UNESP, School of Dentistry, Department of Endodontics, Araçatuba, SP, Brazil

**Keywords:** Radiology, Radiation Protection, Radiation

## Abstract

This study carried out a critical review of the principles of radioprotection, including the ALARA, ALADA, and ALADAIP principles. The Google Scholar and PubMed databases were the search resources, and the following keywords were searched: Linear No-Threshold (LNT); Biologic Effects of Ionizing Radiation (BEIR VII); As Low As Reasonably Achievable (ALARA); As Low As Diagnostically Acceptable (ALADA); As Low As Diagnostically Acceptable, being Indication-oriented and Patient-specific (ALADAIP). This critical review included studies with historical references, reviews, position papers, and clinical and experimental studies. Regarding data extraction, only original articles were selected after the screening process. Much of the current evolution of X-ray machines and radioprotection protocols has stemmed from legitimate concerns about this topic. This development has grown out of the relevant work of organizations like ICRP, UNSCEAR, and other renowned international organizations. Low doses of radiation, such as those used for diagnosis, also occur naturally and are present in everyday life. Although there is no agreement on the actual risk involving low doses, the recommendations of ALARA, ALADA, and ALADAIP prevail, in line with the trend to uphold principles that balance the importance of X-ray diagnostic imaging with the intention of keeping the doses as low as possible. The benefits of X-ray exams, when justified, tend to outweigh the low risks attributed to them.

## Introduction

Deleterious effects have been associated with exposure to high doses of radiation since the early months after Roentgen discovered X-rays in 1895.^1,2^ They are well-known in the scientific literature, and there is a consensus on this topic.^1-3^ However, there is no consensus on the harmful effects of low doses, such as those used for diagnostic imaging.^2,4-6^First of all, it is worth noting that radiation is part of our daily lives. People are constantly exposed to low doses of radiation, also known as background radiation.^2,7^This is related to natural sources, such as radon gas, cosmic radiation, and radioactive elements present in nature. Exposure to this radiation may vary depending on location or even professional activity.^2,3,7^

The estimated risks related to low-dose diagnostic imaging are extrapolated directly from our knowledge about the effects associated with high doses. In this case, there is a linear relationship between the exposure level and radiation-induced cancer. This extrapolation is based on the linear no-threshold (LNT) hypothesis model, and on reports, like the Biological Effects of Ionizing Radiation (BEIR VII Report), which reinforce the concern toward low doses.^2,5,8,9^ However, several authors question the LNT model and BEIR reports due to their limitations in suggesting the risk associated with exposure to low doses of radiation. The LNT model supports the justifications of most principles of radioprotection, including “As Low As Reasonably Achievable” (ALARA), “As Low As Diagnostically Acceptable” (ALADA), and “As Low As Diagnostically Acceptable being Indication-oriented and Patient-specific” (ALADAIP).

An unexpected consequence of spreading information about the risks associated with low-dose radiation is the patients’ fear of undergoing X-ray imaging exams. Sometimes, even health professionals are reluctant to prescribe them. In this context, patients may fail to receive the correct treatment despite the low and unconfirmed risk, and the damage to health may be fatal. Despite the lack of agreement on the deleterious effects associated with low doses of radiation, there is a related concern and consensus on the precaution of using low doses of radiation. This study developed a critical review of the principles of radioprotection, including ALARA, ALADA, and ALADAIP.

## Methods

The Google Scholar and PubMed databases were used with the following search strategy: TOPIC = (Linear No-Threshold) OR TOPIC = (LNT); TOPIC = (Biologic Effects of Ionizing Radiation) OR TOPIC = (BEIR VII) TOPIC = (“As Low As Reasonably Achievable”) OR TOPIC = (ALARA); TOPIC = (“As Low As Diagnostically Acceptable”) OR TOPIC = (ALADA); TOPIC = (“As Low As Diagnostically Acceptable being Indication-oriented and Patient-specific”) OR TOPIC = (ALADAIP). This narrative review was performed with selected historical references, reviews, position papers, and both clinical and experimental studies. Only original articles were selected for data extraction after the screening process.

## Results

In the first decades after the discovery of X-rays and radioactivity, numerous papers were published on the tissue damage caused by high doses of radiation. The number of injuries increased during World War I, because the mobile X-ray machines used in the field were primitive.^1^ This scenario called for devising and setting protocols to guide and standardize radiation use and exposure. In 1925, the International Congress of Radiology (ICR) established the International Commission on Radiation Units and Measurements (ICRU). In 1928, the International Commission on Radiological Protection (ICRP) was established, but was initially named the International X-Ray and Radium Protection Committee (IXRPC).^1^ These international organizations developed many recommendations and standards.

In 1959, the ICRP first mentioned the term “lowest possible,” followed by “as low as practicable,” referring to the use of radiation. In 1977, the concept of “as low as reasonably achievable” (ALARA) was introduced by the ICRP, and has been the main principle of radioprotection ever since.^1,10^ The other precepts are variations of or complement ALARA.^10,11^ The American National Council for Radiation Protection and Measurements (NCRP) suggested modifying the ALARA principle to “As Low As Diagnostically Acceptable” (ALADA).^12^ The DIMITRA European Research Project (Dentomaxillofacial pediatric imaging: an investigation towards low-dose radiation-induced risks) introduced the principle of “As Low As Diagnostically Acceptable being Indication-oriented and Patient-specific” (ALADAIP) as a complement to ALADA.^13^ Table 1 shows the year of creation and the organizations responsible for ALARA, ALADA, and ALADAIP. Although each principle emphasizes a specific aspect, such as the relevance of diagnosis, all focus on lowering patient radiation doses. Hence, ALARA, ALADA, and ALADAIP respect the principles of justification and optimization.^10-15^

**Table 1:** Year of creation and responsible organizations for ALARA, ALADA, and ALADAIP.

Name	Year	Organization
“As Low As Reasonably Achievable” (ALARA)	1977	International Commission on Radiological Protection (ICRP)
As Low As Diagnostically Acceptable” (ALADA)	2014	National Council for Radiation Protection and Measurements (NCRP)
“As Low As Diagnostically Acceptable being Indication-oriented and Patient-specific” (ALADAIP)	2017	DIMITRA Project (Dentomaxillofacial Pediatric Imaging: an investigation into risks induced by low-dose radiation)

According to the justification principle, X-ray exams would be performed only after a clinical examination placed in evidence whether its benefits outweighed its risks.^3,15,16^ The optimization principle regards everything that might reduce radiation exposure during the exam, such as setting the machine collimation to a smaller field of view (FOV), protocols with shorter acquisition time, and use of a protective shield, complemented by a radiation dose that would be as low as reasonably achievable. Another issue is the individual dose limit that must comply with the limits established by national recommendations based on international standards.^3,10,14-17^

The ALADA principle was proposed in 2014 by Dr. Jerrold Bushberg at the NCRP Annual Meeting, focusing on diagnostically acceptable images and the relevance of optimization.^12,15^ According to the ALADA principle, optimization suggests a) selecting X-rays that focus on the individual needs of patients, as opposed to those that are ordered as a routine procedure; b) using the fastest image receptor possible; c) collimating the X-ray beam to expose only the area of interest; d) using thyroid collars; e) child-sizing exposure parameters; f) using cone-beam computed tomography only when necessary.^18^

The DIMITRA group suggested adoption of the ALADAIP principle in 2017, to complement ALADA by adding “Indication-oriented and Patient-specific” (IP) to the acronym. This complementary addition was mainly related to cone-beam computed tomography (CBCT) scans, and the dose for children and adolescents. This was the objective of the DIMITRA group, a European multicenter and multidisciplinary project that is part of the OPERRA research project (Open Project for European Radiation Research Area).^14^

## Discussion

Radioprotection principles have promoted improvements since the first commissions and related organizations.^1^ One of the primary advances in the field of diagnostic imaging was the ongoing evolution of X-ray machines and image receptors, which led to a significant reduction in the dose used to obtain radiographic images in modern X-ray machines.^7^On the other hand, it is increasingly common to find patients who are concerned about undergoing X-ray examinations, and healthcare professionals who are reluctant to prescribe them, as reported in numerous studies. Words like anxiety, fear, and even radiophobia have described the concerns associated with this topic.^5,6,9,19-22^ Furthermore, there are few studies emphasizing the benefits of the exam as opposed to the risks, a sparsity that may explain part of this growing concern.^23^ Therefore, it is vital to understand the potential risk, and compare it with the advantages of X-ray procedures. This information is essential to address the subject as thoroughly as possible.

Low doses of radiation stand below 100 mGy or 100 mSv, and doses above this level are considered high.^5,24^ The effects associated with high doses of radiation (deterministic effects) have been established in the scientific literature, and there is a consensus regarding the topic.^1-3^ However, there is no consensus about the possibility of deleterious effects associated with low doses, such as those used for diagnosis.^5,6,9,19-22^ Estimated risks associated with low-dose diagnostic imaging procedures (stochastic effects) are extrapolated directly from the effects of high doses. It has been posited that there is a linear relationship between radiation exposure levels and radiation-induced cancer.^2,5,8,9^ This extrapolation is based mainly on the linear no-threshold (LNT) hypothesis model, and on reports such as BEIR VII. These concepts originated the theory that there is no safe radiation dose. The concept of proportionality (linearity) in the LNT hypothesis is a mathematical deduction that states that the same amount of radiation exposure shared by any number of people will cause an equal number of cancer cases and deaths. Therefore, the total radiation dose that produces ten cancer cases will do so regardless of whether 10,000 or 100,000 people share it.^25^ Several authors question the LNT model due to its limitations in suggesting the risk associated with exposure to low doses of radiation.^6,9,19-22,26,27^

The LNT model is based on the study by Hermann J. Muller, who received the Nobel Prize for his research.^26^ It has been recently discovered that Hermann J. Muller admitted that another study had strongly challenged his own. According to Calabrese^27^ there are letters from Muller acknowledging the relevance of this disproval of his work, written a few weeks before the Nobel Prize ceremony.^8,27^ The Biologic Effects of Ionizing Radiation (BEIR VII) is the seventh report in a series of publications regarding radiation health effects. The BEIR VII committee used primary sources of data to elucidate the stochastic effects of ionizing radiation, such as occupational radiation studies, medical radiation studies, and atomic bomb survivor studies. The data from this investigation were used with the LNT model to estimate the references for the number of new cancer cases associated with low doses of radiation. It is concerning that BEIR VII should present numerous assumptions for creating these references.^9^

The French Academy found a different conclusion from that of the BEIR VII report. It emphasized the significance of low dose-induced adaptive responses, and suggested that extrapolations from high to low doses could not be considered reliable.^9^ Even the BEIR VII study database rebuts the evidence for deleterious effects at low doses. For example, in the study with atomic bomb survivors, the people exposed to doses below 100 mSv did not bring about an increase in cancer cases, compared to unexposed individuals.^5,19^The Health Physics Society and the American Association of Physicists in Medicine issued the following position statement in 2011, also diverging from the BEIR VII proposal using the LNT model: “The Health Physics Society recommends against quantitative estimation of health risks below an individual dose of 5 rem (50 mSv) in one year, or a lifetime dose of 10 rem (100 mSv) above that received from natural sources. Regarding doses below 5–10 rem (50–100 mSv), risks of health effects are either too small to be observed or nonexistent.”^9,19^

The American Association of Physicists in Medicine (AAPM) issued the statement that: “Risks of medical imaging at patient doses below 50 mSv for single procedures, or 100 mSv for multiple procedures over short periods of time are too low to be detectable, and may be nonexistent. Predictions of hypothetical cancer incidences and deaths in patient populations exposed to such low doses are highly speculative and should be discouraged. These predictions are harmful, because they lead to sensationalistic articles in the public media that cause some patients and parents to refuse medical imaging procedures, thereby placing them at substantial risk by keeping them from receiving the clinical benefits of the prescribed procedures.”^9,19^ In 2021, the AAPM further released the following position statement on medical imaging radiation limits: “The decision to perform a medical imaging exam should be based on clinical grounds, not on the dose from prior imaging-related radiation exposures.”^17^

The United Nations Scientific Committee on the Effects of Atomic Radiation (UNSCEAR) also declared the following: “In general, increases in the incidence of health effects in populations cannot be attributed reliably to chronic exposure to radiation at levels that are typical of the global average background levels of radiation. This is because of the uncertainties associated with the assessment of risks at low doses, the absence of radiation-specific biomarkers for health effects, and the insufficient statistical power of epidemiological studies. Therefore, the UN Scientific Committee does not recommend multiplying very low doses by large numbers of individuals to estimate numbers of radiation-induced health effects within a population exposed to incremental doses at levels equivalent to or lower than natural background levels.”^9^ After several years, the LNT hypothesis and reports like BEIR VII have not been confirmed regarding their conclusions in association with low doses of radiation, further substantiated by the significant evidence of background radiation.

**Figure 1:**
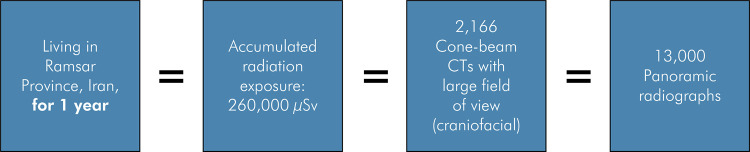
Background radiation from living in Ramsar (Province of Iran) for one year. The accumulated radiation would be equivalent to performing several radiographic exams.7

People are constantly exposed to low doses of radiation from natural sources, also called background radiation,^2,7^ such as radon gas, cosmic radiation, and radiation from the radioactive elements present in nature.^2,3,7^ Hence, low doses of radiation are part of daily life. The technological advancement of X-ray machines and receptors has reduced the doses used for diagnostic purposes to almost natural radiation levels.^7,18^ That is to say, the radiation dose that a person may receive during an X-ray procedure can be directly compared with naturally received doses. Table 2 compares the effective doses delivered by some exams with natural doses received in the United States and Ramsar (Province of Iran). Background radiation exposure may range depending on location or even professional activity. The average background radiation values of the effective dose are about 2 to 4 mSv/y.^28^ Guarapari (Brazil), Kerala (India), Ramsar (Iran), and Yangjiang (China) are areas of high natural background radiation (HNBR). In Ramsar (Province of Iran), the total effective annual dose amounts to 260 mSv/y (260.000 μSv/y), the highest source of background radiation worldwide involving a population.^29,30^ Figure 1 shows that the background radiation accumulated in one year living in Ramsar would be equivalent to performing several radiographic exams. These sites of high background radiation represent sources of information regarding the influence of radiation on exposed populations. Research has shown that, contrary to expectations, the incidence of cancer is no lower in the people living in these areas than in those exposed to X-ray procedures.^28^ In contrast to the LNT hypothesis, low doses of radiation showed a beneficial effect rather than an expected increase in cancer cases.^22,31,32^ According to Sacks et al.^25^ linear assumptions, such as the LNT dose response, are invalid and not protective.

**Table 2:** Typical effective dose from radiographic examinations and equivalent natural radiation (background radiation) in the USA and Ramsar (Iran).

Examination	Median effective dose	Equivalent background exposure	Equivalent background exposure
	(μSv)	(USA)^a^	(Ramsar)^b^
Full-mouth periapical: PSP or F-speed film	200	24 days	6.73 hours
Full-mouth Periapical: CCD sensor (estimated)	100	12 days	3.36 hours
Panoramic	20	2.5 days	40.43 minutes
Cephalometric	5	0.6 day	10.10 minutes
Chest	100	12 days	3.36 hours
Cone-beam CT small field of view (<6 cm)	50	6 days	1.68 hours
Cone-beam CT medium field of view (dentoalveolar, full arch)	100	12 days	3.36 hours
Cone-beam CT large field of view (craniofacial)	120	15 days	4.04 hours
Multidetector CT head	2000	8 months	2.8 days

^a^ References in days are associated with an estimated background radiation of 3.1 mSv/year (ionizing radiation exposure of the United States population).

^b^ References in days are associated with an estimated background radiation of 260 mSv/year (ionizing radiation exposure of the population of Ramsar, Province of Iran).

Source: Mallya SM; Lam EWN. White and Pharoah’s Oral Radiology: Principles and Interpretation. 2018. P 29. Table 3.2; Ghiassi-Nejad M, Mortazavi S, Cameron J, Niroomand-Rad A, Karam P. VERY HIGH BACKGROUND RADIATION AREAS OF RAMSAR, IRAN: PRELIMINARY BIOLOGICAL STUDIES. 2002.

One of the main discussions has been the potential DNA damage that might trigger mutations and cancer. Several studies have presented DNA repair to explain the non-emergence of lesions in patients exposed to low doses of radiation.^21,31,32,33^ However, the dominant paradigm has made researchers overlook hormesis at low-dose-rate ranges, instead of addressing the LNT hypothesis through circular reasoning. Hormesis is a biological phenomenon in which exposure to low or moderate levels of a stressor or toxin can result in beneficial effects or adaptations in an organism. It is characterized by a biphasic dose-response relationship, where a substance or stimulus that is harmful at high doses may be beneficial at low doses.^5,31,32,33^ The hormetic aspect appears when examining broader epidemiological and biological domains.^31,32,33^ The mutation rate related to natural background radiation is very low. There are an estimated three to 30 DNA changes per cell, per year, based on an annual background exposure of 3 mSv in the United States. This is almost 2.5 million times lower than the spontaneous mutation rate from normal metabolism.^25^ Therefore, even if the LNT model of radiation exposure is correct, the adaptive response mechanism that handles spontaneous mutations would make the low additional risk to DNA from the low dose of radiation undetectable.^25^ The DIMITRA group recently conducted research with dental CBCT scans in children, and detected oxidative stress in saliva, but no DNA double-strand breaks in oral mucosa cells.^34^

**Figure 2:**
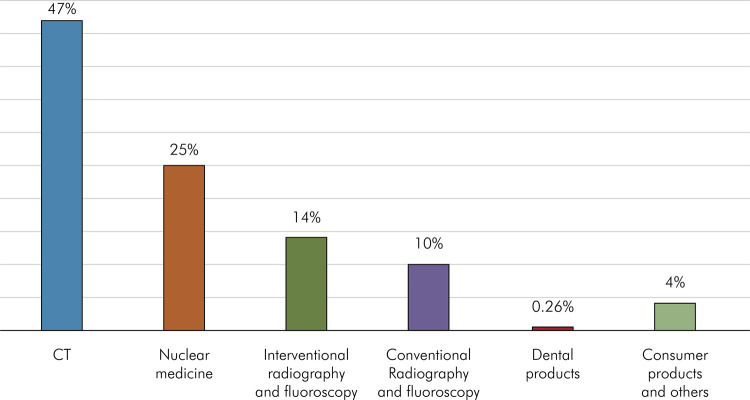
Medical and consumer products and others equivalent to 3.1 mSv/year. In the United States, the radiation dose from medical and consumer products is almost equivalent to the background radiation in a year.7

The estimated risks from dental radiographs are very low. Dental exams contribute minimally to the total radiation rate, compared with medical exams. A demonstration proving whether estimated risks do or do not exist at such low doses is unlikely.^35,36^ Actually in a recent article, the American Academy of Oral and Maxillofacial Radiology established an ad hoc committee, which recommended that patient protective equipment no longer be used in dentomaxillofacial radiography, because the radiation dose to sensitive organs is insignificant and does not pose a significant risk. This applies to exposure of the thyroid gland, breasts, gonads, and fetuses, and to all patients, including pregnant and pediatric patients. Moreover, the use of lead shielding does not significantly reduce a radiation dose, and may lead to potential artifacts.^37^ Figure 2 shows radiation doses from medical, dental, and consumer products in the United States.

In this context, CBCT exams in all fields of dentistry have allowed anticipating problems with more accurate and early diagnoses of changes or diseases in the oral and maxillofacial complex. Professional confidence and assertiveness in clinical decision-making with better prognosis predictability are valuable when indicating this type of imaging exam. However, acquisition protocols must establish sufficient doses to provide exams with good image quality; otherwise, the justification is invalid.

Diagnostic imaging has evolved significantly, and has promoted more accurate diagnoses. Thousands of lives are saved each year because of diagnostic imaging.^23^ Calabrese et al.^9^ summarizes the importance of providing accurate information on this subject: “Our failure to help the public understand the relatively low health risks associated with radiation is now impacting our daily lives and the decisions that people make regarding whether or not to undergo recommended vital imaging procedures that can impact their well-being.”

## Conclusion

Much of the current evolution of X-ray machines and protocols in radioprotection has stemmed from legitimate concerns about this topic. This development has grown out of the relevant work of organizations such as ICRP, UNSCEAR, and other renowned organizations worldwide. Low doses of radiation similar to those used for diagnosis occur naturally in daily life. Although there is no agreement on the actual risk involving low doses, the recommendations of ALARA, ALADA, and ALADAIP prevail, in line with the trend to uphold principles that underscore the significance of exams that favor diagnosis, aiming to maintain doses as low as possible. It is worth noting that the benefits of X-ray exams, when justified, tend to outweigh the low risks attributed to them.
